# Mechanical Aortic Valve Thrombosis During Anticoagulation Using Oral Rivaroxaban: A Case Report

**DOI:** 10.7759/cureus.65007

**Published:** 2024-07-20

**Authors:** Ryoji Kinoshita, Taiju Watanabe, Ryumon Matsumoto, Kazunobu Hirooka

**Affiliations:** 1 Cardiovascular Surgery, Tsuchiura Kyodo General Hospital, Tsuchiura, JPN

**Keywords:** rivaroxaban, warfarin, aortic valve replacement, direct oral anticoagulant, thrombosed mechanical aortic valve

## Abstract

Direct oral anticoagulants (DOACs) are widely used in cardiovascular medicine. Although rivaroxaban has potential benefits for anticoagulation in certain contexts, DOACs remain contraindicated in patients with mechanical heart valves. This case report highlights the life-threatening risks of rivaroxaban use in patients with mechanical aortic valves, underscoring the lack of proven efficacy and the necessity of adhering to established anticoagulation protocols with warfarin for this patient population. Here, we report a case of a 65-year-old man who had previously undergone aortic valve replacement and developed a thrombus in the mechanical aortic valve six months after switching from warfarin to rivaroxaban. The patient experienced a sudden loss of consciousness and chest discomfort. Echocardiography revealed a thrombus in the valve requiring urgent reoperation and replacement with a bioprosthetic valve. The postoperative recovery was uneventful.

## Introduction

In cardiovascular medicine, direct oral anticoagulants (DOACs) are not only indicated for non-valvular atrial fibrillation, deep vein thrombosis, and pulmonary embolism but also for valvular atrial fibrillation [1] and atrial fibrillation after bioprosthetic valve implantation [2]; however, they remain contraindicated for anticoagulation after mechanical valve implantation [3,4]. Several cases of thrombosed mechanical valves have been reported in patients receiving DOACs; however, most cases involved mechanical mitral valves [5,6], and to the best of our knowledge, reports on the use of rivaroxaban following mechanical aortic valve replacement are lacking. Herein, we report a case of thrombosis in the mechanical aortic valve that required reoperation after a change in medication from warfarin to rivaroxaban.

## Case presentation

**Figure 1:**
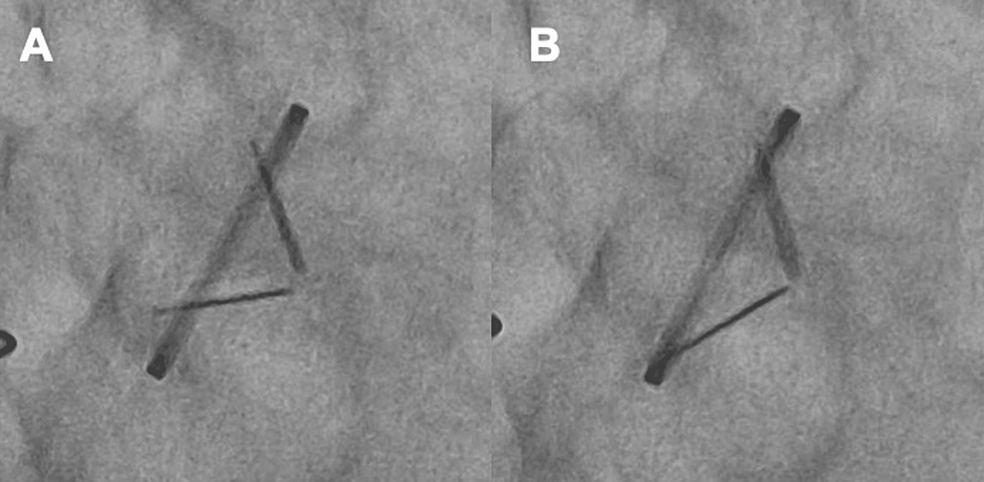
Preoperative cinefluoroscopy. Cinefluoroscopy showed both opening and closing restrictions were occurring. (A) The mechanical valve opening angle is 51 degrees. (B) The mechanical valve closing angle is 37 degrees.

**Figure 2:**
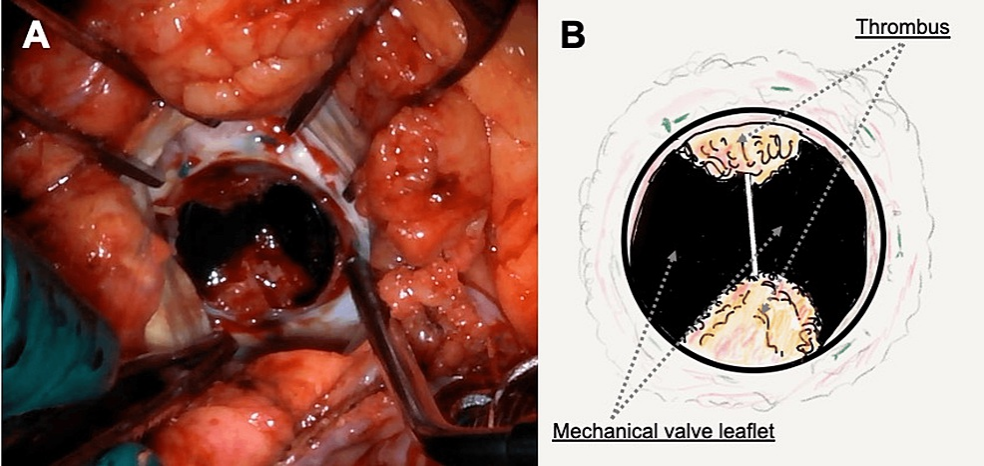
Thrombosed mechanical aortic valve. (A) Intraoperative findings. Following the aortotomy, the implanted mechanical valve and the thrombus formed on its surface are observed. (B) Schema representation of intraoperative findings. * This illustration is an original creation by the authors.

A 65-year-old man with a history of aortic valve replacement (Regent™ mechanical aortic valve, 21 mm; St. Jude Medical, Saint Paul, MN) at our hospital for severe aortic regurgitation 10 years prior sought emergency care at another hospital owing to sudden loss of consciousness and subsequent chest discomfort. The patient had been under the care of our surgical team for anticoagulation management with warfarin for approximately six months post surgery, during which we monitored and adjusted his medication to achieve adequate prothrombin time (PT) prolongation. After this period, the responsibility for managing the patient’s anticoagulation therapy was transitioned to his primary care physician; however, obtaining an optimal PT prolongation has been difficult since the primary care physician started to manage warfarin medication in the outpatient setting. Approximately six months prior to presentation, bucolome was added to his regimen by his primary care physician, which resulted in excessive PT prolongation and intramuscular bleeding in the lower limbs. Consequently, warfarin was discontinued for a period of 13 days, and rivaroxaban 15 mg was initiated. At the time of initiating rivaroxaban therapy, the patient’s prothrombin time-to-international normalized ratio (PT-INR) was measured at 0.99. On presentation at the emergency department, echocardiography revealed a highly echogenic mass around the mechanical aortic valve, reduced leaflet mobility, central valve regurgitation, and accelerated transvalvular flow, indicating mechanical aortic valve dysfunction (Videos 1-3). Cinefluoroscopy revealed valve opening and closing angles of 51° and 37°, respectively, indicating both opening and closing restrictions (at the fully open and closed positions, the Regent™ mechanical aortic valve has leaflet angles of 85° and 25°, respectively) (Video 4 and Figure 1). Based on a diagnosis of prosthetic valve dysfunction due to a thrombus with loss of consciousness, continuous intravenous heparin was started, but the thrombus showed no signs of shrinking. Consequently, the patient was transferred to our hospital for early re-operation. The results of the transthoracic echocardiogram performed at our hospital were as follows: left ventricular diastolic diameter, 47 mm; left ventricular systolic diameter, 29 mm; left ventricular ejection fraction, 67%; transaortic maximum velocity, 4.2 m/sec; transaortic maximum/mean pressure gradient, 71/43 mmHg; and moderate-severe transvalvular regurgitation. On the fourth day after transfer, the patient underwent cardiac surgery again via re-sternotomy. After establishing a cardiopulmonary bypass, the aorta was cross-clamped, and cardiac arrest was achieved. Inspection of the implanted mechanical valve revealed a thrombus attached to the hinge of the valve shaft that hindered valve movement (Figure 2). The mechanical valve was removed, and considering the patient’s age, a bioprosthetic valve (INSPIRIS, 21 mm; Edwards Lifesciences, Irvine, CA) was implanted in the supra-annular position (Video 5). After bioprosthetic valve implantation, a PT-INR ratio of approximately 2 was easily achieved using 3-3.5 mg warfarin under appropriate nutritional and medication counseling. The postoperative course was uneventful, and the patient was discharged home on postoperative day 11. Warfarin was continued for three months after bioprosthetic valve implantation, and anticoagulant medication was completely discontinued thereafter.

**Video 1:** Preoperative transthoracic echocardiography, long axis view. * The source of this video is attributed to the authors.

**Video 2:** Preoperative transthoracic echocardiography, short axis view. * The source of this video is attributed to the authors.

**Video 3:** Preoperative transthoracic echocardiography, color Doppler mode on long axis view. * The source of this video is attributed to the authors.

**Video 4:** Preoperative cinefluoroscopy. * The source of this video is attributed to the authors.

**Video 5:** Surgical findings video. * The source of this video is attributed to the authors.

## Discussion

Unlike warfarin, which exerts anticoagulant effects via vitamin K antagonism, DOACs achieve anticoagulation by directly inhibiting specific coagulation factors. The commonly used DOACs are dabigatran, which directly inhibits thrombin, and rivaroxaban, apixaban, and edoxaban, which directly inhibit coagulation factor Xa. Various studies have demonstrated that DOACs are non-inferior to warfarin in terms of preventing embolic events and reducing the risk of bleeding events [7-9].

DOACs are clinically favored owing to their fixed-dose usage without the need for dose adjustment for each patient, lack of requirement for regular blood tests, and minimal dietary restrictions, which are preferred by patients. Moreover, DOACs are effective in patients with atrial fibrillation undergoing bioprosthetic valve implantation. Furthermore, subgroup analyses in randomized controlled trials have shown that dabigatran, apixaban, and edoxaban are non-inferior to warfarin in patients with atrial fibrillation undergoing bioprosthetic valve implantation [10-12], and a meta-analysis of 241 randomized controlled trials showed that no DOAC is inferior to warfarin for anticoagulation in patients with atrial fibrillation undergoing bioprosthetic valve implantation [13].

However, the efficacy of DOACs for anticoagulation after mechanical valve implantation has not yet been proven. Previously, DOACs were expected to be alternatives to warfarin for anticoagulation in patients with mechanical valve implantation. However, in the RE-ALIGN trial (2012), a clinical study on dabigatran administration in patients undergoing mechanical heart valve replacement, the dabigatran group experienced significantly more thromboembolic and bleeding events than the warfarin group. Consequently, the RE-ALIGN trial was terminated in 2013 [14].

Few small-scale studies with observation periods of less than six months have indicated the efficacy of rivaroxaban for anticoagulation after mechanical valve implantation [15-17]. However, the evidence is insufficient to support the clinical use of rivaroxaban, and further large-scale studies are required.

Theoretically, the risk of embolic events is lower after aortic valve replacement than after mitral valve replacement because of high-pressure blood flow throughout the left ventricular outflow tract, which makes thrombus formation less likely [18]. Therefore, alternatives to warfarin for anticoagulation in patients undergoing aortic valve replacement are needed, and trials are being conducted to verify the efficacy of antiplatelet agents alone or in combination with DOACs [19,20].

However, despite using a lower dose of rivaroxaban than previously tested, our patient developed a thrombus in the aortic valve approximately six months after switching to rivaroxaban. This suggests that even at the currently approved dosages, rivaroxaban may pose a risk of thrombotic complications in mechanical aortic valves, leading to severe prosthetic valve dysfunction. Although higher doses of rivaroxaban might be effective, concerns about the increased risk of bleeding events indicate that robust large-scale clinical trials are necessary before the indications of rivaroxaban are extended to include anticoagulation in patients after mechanical valve implantation.

In this patient, the importance of warfarin therapy for anticoagulation after mechanical valve implantation was not well-recognized by the primary care physician, patient, or family. Therefore, education of primary care physicians, patients, and their families regarding the need for warfarin and the lack of alternative anticoagulation is imperative. The previous physician found it challenging to adjust the anticoagulant effect of warfarin. However, the effectiveness of warfarin can be influenced by interactions with dietary content and drugs, which makes adjustments challenging, and we achieved control within the target range using standard dosages through appropriate dietary and medication guidance.

While enoxaparin and low molecular weight heparin (LMWH) are recommended as bridging treatments in cases of thrombotic complications, we initiated continuous intravenous heparin infusion immediately after the patient presented with a thrombus. Despite this intervention, the thrombus did not reduce in size, prompting us to proceed with relatively urgent reoperation to replace the dysfunctional mechanical valve with a bioprosthetic valve. This case highlights the necessity of timely surgical intervention when anticoagulation therapy alone is insufficient to resolve thrombotic events.

## Conclusions

DOACs are presently contraindicated for anticoagulation after mechanical valve implantation due to the risk of thrombotic complications. In the present case, switching from warfarin to rivaroxaban, an off-label use, resulted in thrombosis in the mechanical aortic valve, necessitating reoperation within approximately six months. This case underscores the importance of adequate anticoagulation using warfarin for patients with mechanical prostheses. While LMWH is often used as a bridging therapy, even continuous intravenous heparin was ineffective in this patient after a thrombotic event, requiring urgent reoperation. Healthcare providers must adhere to current guidelines advocating warfarin use after mechanical valve implantation and ensure that patients and their families are educated about the critical importance of maintaining appropriate anticoagulation therapy.
